# Thermal remodelling of Alternanthera mosaic virus virions and virus-like particles into protein spherical particles

**DOI:** 10.1371/journal.pone.0255378

**Published:** 2021-07-28

**Authors:** Tatiana I. Manukhova, Ekaterina A. Evtushenko, Alexander L. Ksenofontov, Alexander M. Arutyunyan, Angelina O. Kovalenko, Nikolai A. Nikitin, Olga V. Karpova

**Affiliations:** 1 Faculty of Biology, Department of Virology, Lomonosov Moscow State University, Moscow, Russia; 2 Belozersky Institute of Physico-Chemical Biology, Lomonosov Moscow State University, Moscow, Russia; Panjab University Chandigarh, INDIA

## Abstract

The present work addresses the thermal remodelling of flexible plant viruses with a helical structure and virus-like particles (VLPs). Here, for the first time, the possibility of filamentous Alternanthera mosaic virus (AltMV) virions’ thermal transition into structurally modified spherical particles (SP) has been demonstrated. The work has established differences in formation conditions of SP from virions (SP_V_) and VLPs (SP_VLP_) that are in accordance with structural data (on AltMV virions and VLPs). SP originate from AltMV virions through an intermediate stage. However, the same intermediate stage was not detected during AltMV VLPs’ structural remodelling. According to the biochemical analysis, AltMV SP_V_ consist of protein and do not include RNA. The structural characterisation of AltMV SP_V/VLP_ by circular dichroism, intrinsic fluorescence spectroscopy and thioflavin T fluorescence assay has been performed. AltMV SP_V/VLP_ adsorption properties and the availability of chemically reactive surface amino acids have been analysed. The revealed characteristics of AltMV SP_V/VLP_ indicate that they could be applied as protein platforms for target molecules presentation and for the design of functionally active complexes.

## Introduction

Alternanthera mosaic virus (AltMV) is a member of the genus *Potexvirus*, family *Alphaflexiviridae* [1]. A previously characterised strain, AltMV-MU (Moscow University) (accession number FJ822136 in the GenBank), was used [2]. AltMV-MU (further AltMV) virions are helical filamentous particles 570 nm in length and 13 nm in diameter, consisting of RNA and coat protein (CP). AltMV CP self assembles *in vitro* in the absence of viral RNA into extended polymers (virus-like particles, VLPs) whose length varies within the range 60–2000 nm [3]. According to our earlier results [4], despite AltMV VLPs and virions having a morphological similarity, they differ in structure, as revealed indirectly by trypsin hydrolysis and then confirmed by cryoelectron microscopy. Firstly, the diameter of AltMV VLPs (152 Å) is larger than AltMV virions (135 Å). Also, the number of CP subunits per turn is greater for VLPs (~9.55 compared to 8.75 in virions), while the pitch of the helix is the same for both types of particle (~35.7 Å). The inner channel of VLPs is wider (~30 Å for VLPs and 20 Å for virions) due to the absence of viral RNA [4]. Previously, we reported that some helical plant viruses (tobacco mosaic virus (TMV), potato virus X (PVX)) can generate structurally modified spherical particles (SP) after heating at 94°C or 90°C, respectively. TMV and PVX SP contain only thermally denatured CP molecules and lack viral RNA [5,6]. It has been established that TMV SP diameter depends on initial virus concentration and varies from 50 nm upwards [5]. At the same time, such a clear dependence was not detected between initial virus concentration and the size of SP formed from PVX [6]. We found that the structure of the protein in TMV SP and PVX SP differs markedly from that in native virions. One of the most distinctive features of SP protein is the low proportion of α-helix structures and the considerable percentage of β- and disordered structures, in contrast to the protein within intact virions [6,7]. We also demonstrated that TMV SP are highly stable in the context of various factors (temperature, storage, centrifugation) and possess unique adsorption properties [5,8–11]. Additionally, it has been revealed that the virions to SP transition is accompanied by changes in the composition of surface amino acids which apparently affect adsorption capacity [12]. TMV SP demonstrate great potential for various biotechnology applications. We revealed the effective adjuvant properties of TMV SP, which have enabled their implementation in the development of a candidate vaccine against rubella [11]. Furthermore, it has been shown that TMV SP elicits a potent antitumour immunity, slows tumour growth and increases survival time [13]. TMV SP are able to form complexes with chelated paramagnetic gadolinium that provide a perspective contrast agent for magnetic resonance imaging [14]. Additionally, it has been established that TMV SP can bind gold nanoparticles [15] and could be applied as a biotemplate for the linking of silver nanoparticles [16] that can serve as a basis for developing new approaches to antiviral [17] and antibacterial treatment [18]. AltMV was chosen to continue the study of helical plant viruses’ thermal remodelling and the properties of formed SP. As we reported earlier [19], it is possible to obtain SP from AltMV VLPs (SP_VLP_) under conditions suitable for PVX and TMV SP formation. However, SP were not obtained from AltMV virions (SP_V_) under such experimental conditions. Therefore, it was intriguing to find conditions appropriate for deriving SP_V_ and to compare the properties of SP_V_ and SP_VLP_.

## Materials and methods

### Virus purification and coat protein isolation

AltMV-MU viral strain was accumulated in *Nicotiana benthamiana* and isolated according to an existing protocol for AltMV purification using a Himac CP100WX ultracentrifuge (Hitachi, Hitachinaka City, Japan) [4]. AltMV CP was isolated using the method of salt deproteinisation with 2M LiCl, as described previously [2,20].

### RNA extraction

To assess whether RNA was present in SP_V_, samples obtained by a protocol described below were precipitated. Precipitation was carried out by low-speed (700 g) centrifugation, using a Microspin FV-2400 (BioSan, Riga, Latvia) for 5 min. The resulting pellet of SP_V_ was resuspended in Milli-Q water (Simplicity UV; Millipore, Billerica, USA) and was used for RNA isolation according to [21].

### AltMV SP generation

SP_V_ and SP_VLP_ were generated by virions/VLPs being heated to 98°C for 10 sec (experiments characterising stages of thermal transition into SP_V_ and SP_VLP_) or 30 sec (in other cases) in the Tercyc thermocycler (DNA Technology, Moscow, Russia). SP_V_ were obtained in 0.15 M NaCl solution, while SP_VLP_ were generated in Milli-Q water.

### Transmission electron microscopy

Samples were prepared using standard procedures [4] and observed using a transmission electron microscope, JEOL JEM-1400 TEM (JEOL, Tokyo, Japan). Images were recorded on an Olympus Quemesa digital camera, using iTEM software (Olympus Soft Imaging Solutions GmbH, Munster, Germany). SP diameter was measured using an image processing program designed for scientific multidimensional images, ImageJ (National Institutes of Health, Bethesda, Maryland, USA). SP sizes are presented as the mean ± SD.

### Electrophoresis analysis

RNA analysis was performed in 1% agarose gel, according to [22]. Proteins were studied by electrophoresis in SDS-PAGE (8–20%), as described in [5]. Gels were imaged and analysed using the ChemiDoc™ XRS+ System, with Image Lab™ Software (Bio-Rad Laboratories, Hercules, California, USA).

### Spectra measurements

Milli-Q water was used in the case of AltMV VLPs and SP_VLP_, 15 mM NaCl solution was used for SP_V_ and 12.5 mM Tris-HCl pH 8.0 was used in the case of AltMV virions for far-UV CD and intrinsic fluorescence measurements. CD spectra in the 200–260 nm region were recorded in 1–2 mm cells at 25°C, using a Chiroscan CD spectrometer (Applied Photophysics, Surrey, UK). Sample concentrations were in the range of 0.1–0.3 mg/ml. The spectra were recorded at a speed of 0.5–1.0 nm/s with baseline subtraction. The spectra measured were smoothed using the instrument software Pro Data. The [ϴ] values were calculated taking the mean molecular weight of amino acid residues to be 110. The intrinsic fluorescence spectra were recorded in 1 cm cells, using a FluoroMax spectrofluorometer (HORIBA Jobin Yvon, Edison, NJ, USA), at 25°C. Fluorescence was excited at 280 nm and the emission spectra were recorded in the 300–400 nm range.

### Thioflavin T (ThT) fluorescence assay

AltMV virions, VLPs and SP_V/VLP_ were mixed with freshly prepared ThT (Sigma-Aldrich, St. Louis, MO, USA) according to [23]. The fluorescence emission spectra were measured between 450 and 620 nm, with excitation at 445 nm, using a FluoroMax spectrofluorometer. The background ThT fluorescence was measured using ThT buffer only.

### Fluorescence labelling

The procedure of fluorescein isothiocyanate (FITC) (Sigma-Aldrich, St. Louis, MO, USA, 3326-32-7) labelling of AltMV virions, VLPs and SP_V/VLP_ was carried out in accordance with the manufacturer’s protocol, with some modifications. The initial concentration of the samples before labelling was at least 1 mg/ml. Labelling was performed in 0.1 M sodium carbonate buffer, pH 9. To 1 ml of samples, 50 μl of FITC solution (1 mg/ml) was added and then samples were incubated in the dark, overnight, at 4°C. To remove unbound FITC, the labelled samples were dialysed for 4 h against phosphate-buffered saline (PBS) and for 1 h against Milli-Q water. The 5-(N-Maleimido)-fluorescein diacetate (Chem-impex, Wood Dale, USA, 22988) labelling procedure was carried out in accordance with [24]. The results of labelling were detected by fluorescence microscopy, using an Axiovert 200M fluorescence microscope (Carl Zeiss, Gottingen, Germany) equipped with a digital cooled camera ORCAII-ERG2 (Hamamatsu Photonics K.K, Hamamatsu City, Japan), and by SDS-PAGE electrophoresis with subsequent analysis in UV light using the ChemiDoc™ XRS+ System.

## Adsorption properties study

100 μg of SP_V/VLP_ were incubated with 10 μg of model antigen, (recombinant protein containing repeated sequences of influenza virus A M2e epitope, MW 24 kDa), in water and at room temperature, overnight. Then, the complexes were centrifuged at Microspin FV-2400 for 5 min to remove unbound proteins. The sedimented complexes were resuspended in Milli-Q water and prepared for immunofluorescence microscopy according to a protocol described previously [9]. For complexes detection, an Axiovert 200M fluorescence microscope equipped with a digital cooled camera, ORCAII-ERG2, was used.

## Data analysis

SP size statistical differences were analysed using one-way ANOVA with a *post hoc* Tukey HSD test. Probability values (p values) of less than 0.05 were considered to be significant. The data are presented as mean ± SD.

CD spectra were recorded three to five times, averaged and smoothed using the instrument software Pro Data. Two independent experiments were carried out. The data of the secondary structure content are calculated as the mean ± SEM of two experiments.

In the case of intrinsic fluorescence and ThT assay, three independent experiments were conducted. Fluorescence maxima are calculated as the mean ± SEM.

## Results and discussion

Previously, it was demonstrated that the heating of AltMV virions under conditions suitable for PVX and TMV virions did not result in the generation of AltMV SP_v_ [19]. To identify specific conditions that are suitable for AltMV virions’ transformation to SP_v_, an attempt was made to heat virions to 98°С, in various buffers (S1 Fig). Particles similar to SP from PVX were generated after treatment in PBS pH 7.4 (S1A Fig). In other conditions, only irregular structures that had a tendency to aggregate were detected (S1B–S1D Fig). It became apparent that the key component of PBS determining AltMV SP_V_ formation is 0.15 M NaCl (S1F Fig). After the same treatment in 0.01 М phosphate buffer, рН 7.4, no SP_V_ were detected (S1E Fig). However, according to our earlier results [19], AltMV VLPs successfully transform into SP_VLP_ after heating in Milli-Q water. Apparently, SP_V_ and SP_VLP_ form in different conditions, because of the differences in virions’ and VLPs’ structural characteristics, including RNA-protein interactions in virions and the absence of RNA in VLPs [4].

**Fig 1:**
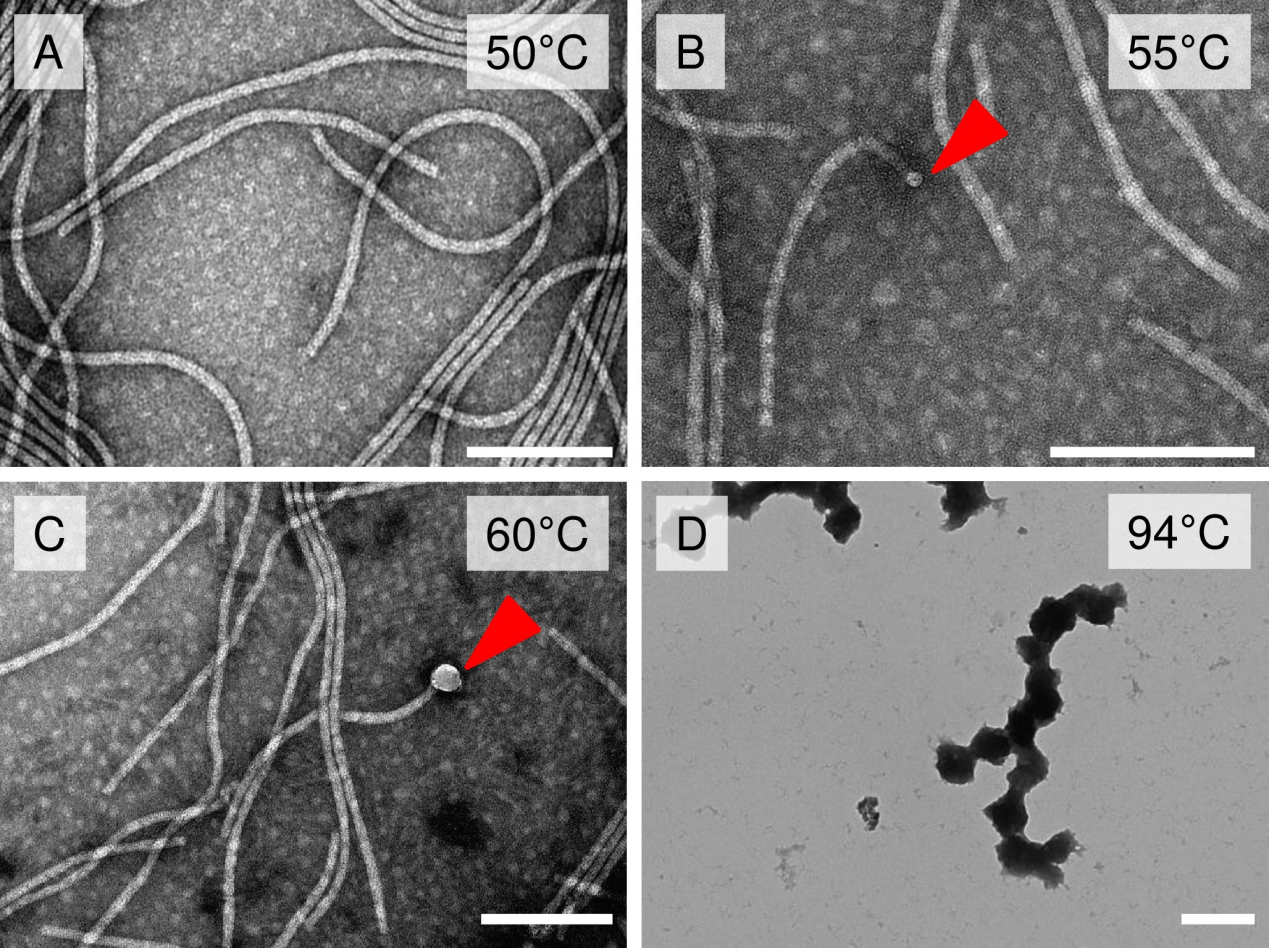
The stages of thermal transition during the formation of SP from AltMV virions. **A)** AltMV virions after thermal treatment at 50°C; **B)** intermediate form, thermal treatment at 55°C; **C)** intermediate form, thermal treatment at 60°C; **D)** SP_V_ formed after thermal treatment at 94°C. Transmission electron microscopy, staining with 2% uranyl acetate. Arrows indicate the swelling at the end of the virion (intermediate form). Thermal treatment was carried out for 10 sec. Bars are 200 nm in A, B, C; bar is 400 nm in D.

Here, several temperature points of the structural transition of AltMV virions and VLPs into SP were analyzed. AltMV virions were incubated at different temperatures (50°C, 55°C, 60°C, 94°C) for 10 sec. At 50°C, only unmodified AltMV virions were detected (Fig 1A). However, heating to 55°C led to swelling at one end of the virion (Fig 1B). This modified state of virions represents the intermediate form (IF). Upon further heating to 60°C, in addition to the IF referred to in Fig 1B, there was the appearance of IF with greater thickening at the end of the virion (Fig 1C). Subsequent heating to 94°C resulted in mature SP_V_ formation (Fig 1D). There were no visible differences in SP_V_ morphology at either 94°C or 98°C (S1F Fig).

Therefore, the temperatures required for structural transformation are not similar for two flexible filamentous potexviruses (AltMV and PVX). The AltMV IF was observed at a lower temperature (55°C for AltMV) than the PVX (70°C); while mature AltMV SP_V_ were produced after heating at 94°C, mature PVX SP were detected at 90°C [6]. It is assumed that structural transition temperature may depend on buffer composition. AltMV SP_V_ need 0.15 М NaCl, while PVX SP form in Milli-Q water or in 0.01 M Tris-HCl, pH 7.8. This hypothesis is consistent with data that suggest that NaCl may increase proteins’ thermal stability [25]. Differences in the SP formation conditions of two morphologically similar helical viruses (AltMV and PVX) belonging to the same genus will demonstrate the impact of structural viruses’ features on the thermal remodelling.

**Fig 2:**
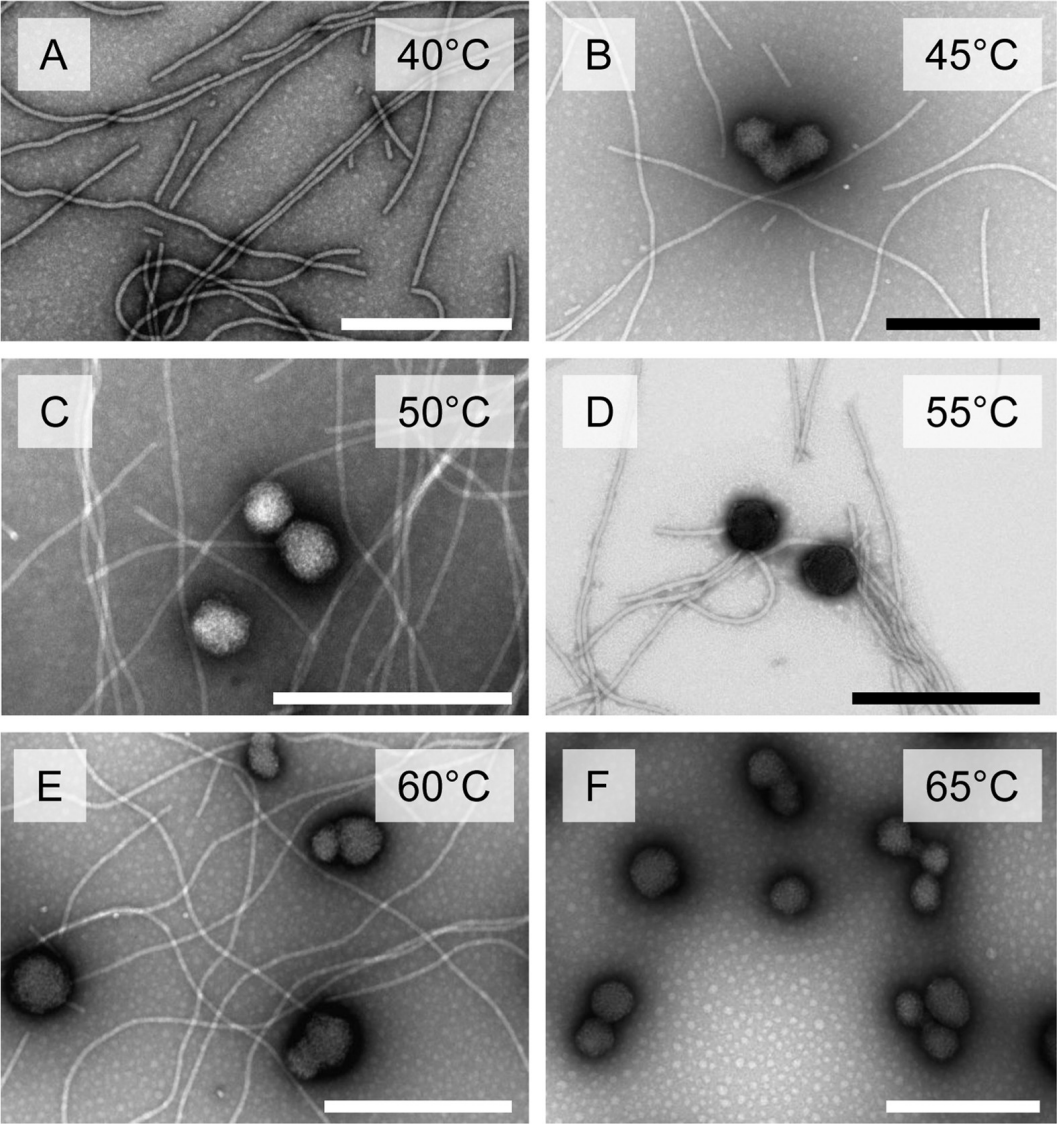
The stages of thermal transition during the formation of SP_VLP_. **A)** Thermal treatment of AltMV VLPs at 40°C; **B), C), D), E)** thermal treatment of AltMV VLPs at 45°C, 50°C, 55°C and 60°C, respectively; **F)** SP_VLP_ formed after thermal treatment of AltMV VLPs at 65°C. Transmission electron microscopy, staining with 2% uranyl acetate. Thermal treatment was carried out for 10 sec. Bars, 500 nm.

The process of structural remodelling for SP_VLP_ was also studied. No IF was detected similar to virions with a swollen end. At 40°C, morphologically stable VLPs were observed (Fig 2A). VLPs heated to 45°C resulted in partial SP_VLP_ formation, but some VLPs stayed in unmodified filamentous form (Fig 2B). Upon a further increase in the temperature to 60°C, both SP_VLP_ and VLPs were detected (Fig 2C–2E). Heating to just 65°C resulted in all VLPs transforming into SP_VLP_ (Fig 2F).

According to our earlier results the size of SP from TMV depends on initial virus concentration [6]. Increasing the initial concentration of TMV leads to the formation of SP with a larger diameter. However, for SP from PVX and barley stripe mosaic virus, the size dependence of the particles formed on the initial concentration of the virus was not so clear [6,19].

AltMV SP_V_ were obtained with initial concentrations 0.1, 1, 2 and 5 mg/ml. AltMV with a concentration of 0.1 mg/ml formed SP with a mean diameter of 124 ± 31 nm. Increasing virus concentration to 1 mg/ml led to the formation of SP with a mean diameter of 160 ± 48 nm. A further increase of initial virus concentration to 2–5 mg/mL had no substantial effect on the particles’ size (S2 Fig).

It is important to note that, after the heating of different AltMV VLPs samples (obtained during different AltMV CP isolation) with the same concentration, SP_VLP_ with various mean diameters were obtained. In our previous work [19], we reported that the average diameter of SP_VLP_ obtained from AltMV VLPs at a concentration of 0.1 and 1.0 mg/ml was 100 nm. In the present study, preparations of SP_VLP_ with a mean diameter of 70 ± 10 nm, 118 ± 13 nm and 157 ± 28 nm, from 1.0 mg/ml of AltMV VLPs, were obtained. SP_VLP_ size probably depends on the efficiency of AltMV VLPs’ assembly. It is not clear which factors impact on the efficiency of AltMV CP polymerisation into VLPs, however; this aspect requires additional research.

**Fig 3:**
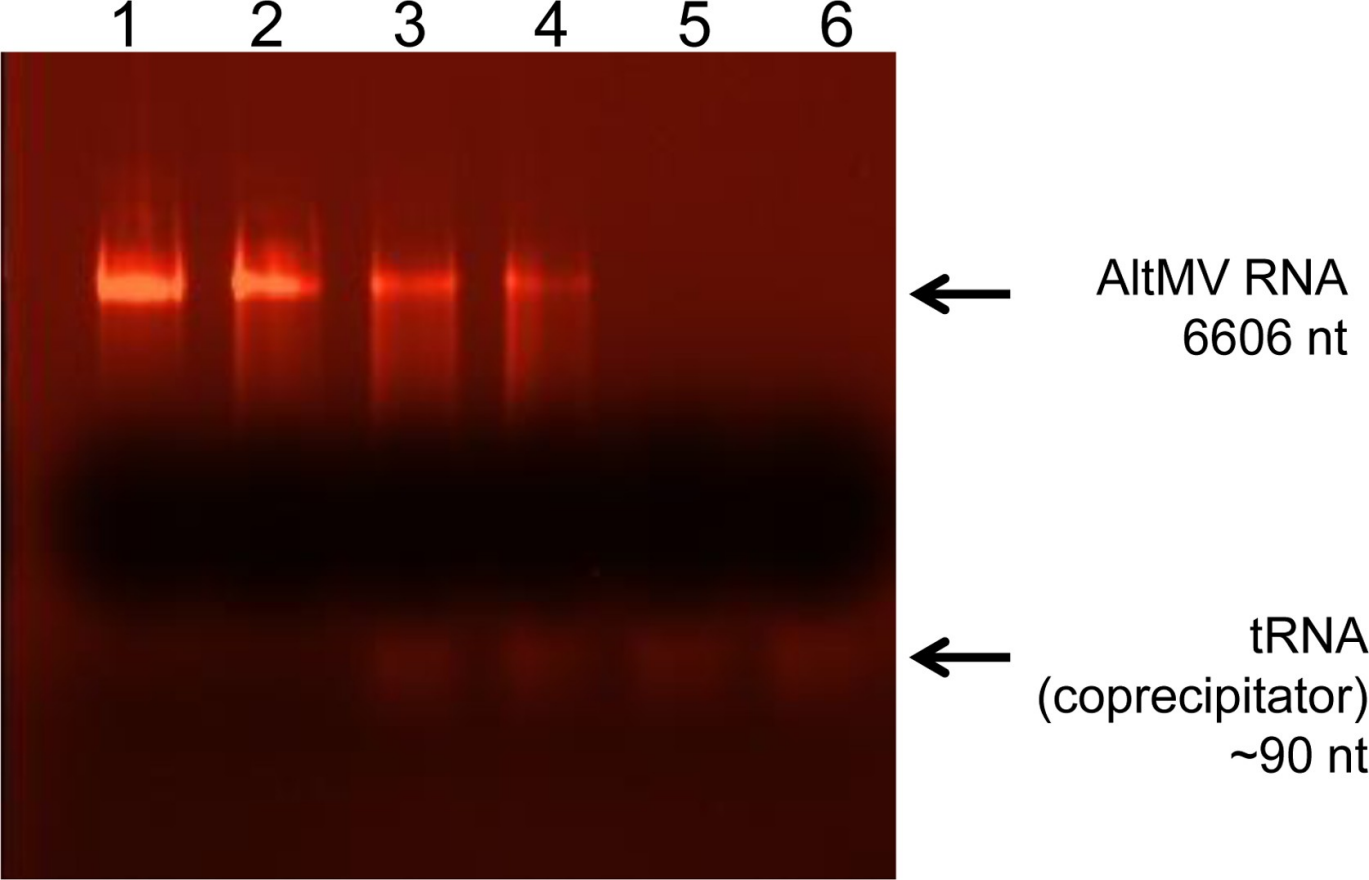
Characterisation of the RNA state within the AltMV SP_V_. **1)** AltMV RNA (positive control)– 6606 nt; **2)** RNA isolated from AltMV virions (sample 4); **3)** RNA isolated from non-centrifuged AltMV SP_V_ (sample 3); **4)** RNA isolated from supernatant after SP_V_ low-speed centrifugation (sample 2); **5)** RNA isolated from SP_V_ pellet after low-speed centrifugation (sample1); **6)** negative control (Milli-Q water passed through all procedures similar to samples). Virions were heated to 98°C for 30 sec. Electrophoretic analysis in 1% agarose gel. The gel was stained with ethidium bromide.

To analyse the biochemical component elements of AltMV SP_V_, the following experiment was conducted. AltMV SP_V_ with a concentration of 1 mg/ml were precipitated by low-speed centrifugation, as described in Materials and Methods. The SP_V_ pellet was resuspended in Milli-Q water and was used for RNA isolation (sample 1). Also, RNA was extracted from supernatant (sample 2), from intact SP_V_ preparation which had not been centrifuged (sample 3) and from an equal quantity of untreated AltMV virions (sample 4). No viral RNA was detected in sample 1 (Fig 3, lane 5). At the same time, residual non-degraded RNA was observed in samples 2 and 3 (Fig 3, lane 4 and 3, respectively). It is worth emphasising that these samples were obtained by heating AltMV virions to 98°C for 30 sec. Nevertheless, some part of RNA was preserved intact after such thermal treatment. This RNA could be detected if the sample was not precipitated (Fig 3, lane 3) and SP_V_ were not separated from supernatant. Remarkably, previously, in the case of PVX and TMV SP, complete RNA degradation was demonstrated upon heating at 94-98°C for 10 sec [5,6]. Despite the presence of RNA in the non-centrifuged sample (Fig 3, lane 3), no RNA was detected in SP_V_ that were purified from supernatant (Fig 3, lane 5). Thus, AltMV SP_V_, as well as those of TMV and PVX, consisted exclusively of thermally denatured CP and did not contain viral RNA.

SP_V_ and SP_VLP_ protein component was also analysed. No degradation or fragmentation of AltMV CP occurred upon the heating of virions and SP formation from AltMV virions (S3 Fig, lane 4). The same results were obtained for SP_VLP_ (S3 Fig, lane 6).

**Fig 4:**
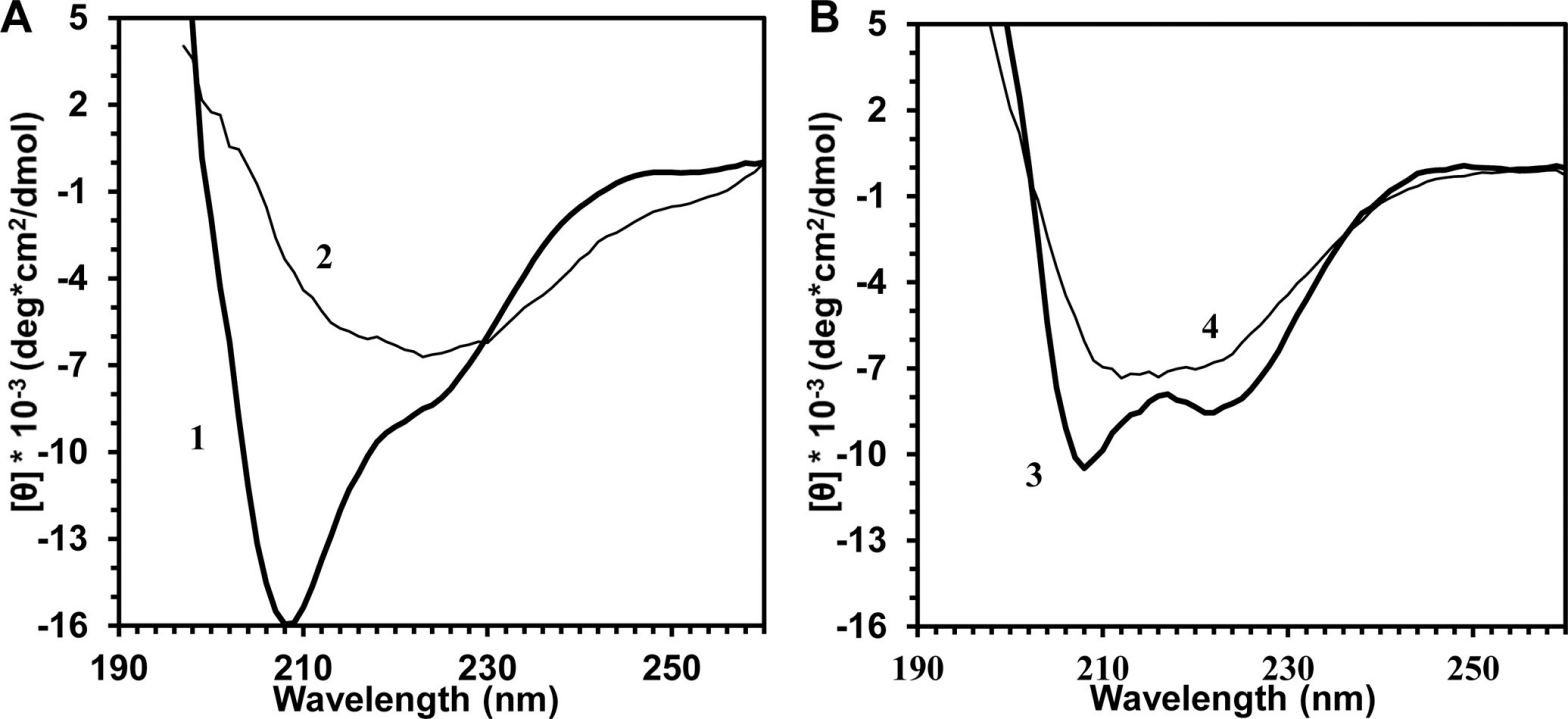
Far-UV CD spectra of **A)** AltMV virions (1) and SP_V_ (2); **B)** AltMV VLPs (3) and SP_VLP_ (4). The initial virus and VLPs concentration was 1 mg/ml. The spectra were recorded at 25°С. The solution details are specified in Materials and Methods. Two repeated experiments were carried out with similar results. The spectra presented in the figure are the visualization of one repetition.

Previously, we have reported considerable changes in the CP structure of other helical viruses (TMV, PVX) after thermal transition to SP [6,7]. In the current work, similar structural shifts after thermal remodelling of AltMV virions and VLPs were observed. For this purpose, circular dichroism (CD), intrinsic fluorescence spectroscopy and thioflavin T (ThT) assay were used. The CD spectra of AltMV virions, VLPs, SP_V_ and SP_VLP_ (Fig 4) were processed using program K2D3. The prediction of secondary structure is presented in Table 1.

**Table 1:** Comparison of secondary structure content in protein in AltMV VLPs and AltMV SP_V/VLP_.

	α-helices, %	β-structure, %	unordered, %
AltMV VLPs [26]	27,5	17	56,5
AltMV SP_VLP_^a^	10±3	35±1	54±2
AltMV SP_V_^a^	3±1	42±1	55±1

^a^Data for SP_VLP_ and SP_V_ are expressed as the mean ± SEM of two replicates.

The analysis of the data obtained confirmed that, similar to SP from TMV and PVX, AltMV SP_V_ and SP_VLP_ formation was accompanied by alteration of secondary structure. The content of α-helices in CP in AltMV virions and VLPs was higher than in protein within both types of SP. At the same time, the proportion of β-structure increased after thermal treatment.

**Fig 5:**
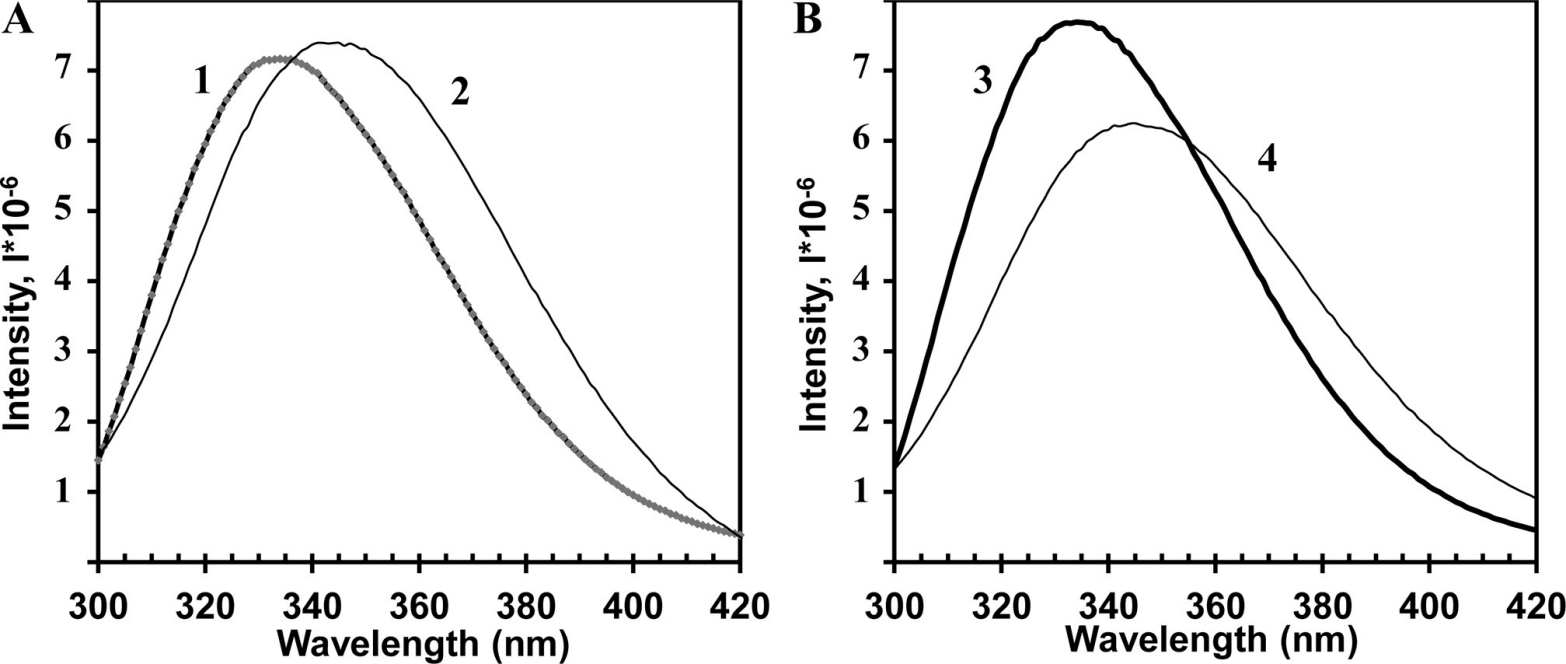
Intrinsic fluorescence spectra of **A)** AltMV VLPs (1) and SP_VLP_ (2); **B)** AltMV virions (3) and SP_V_ (4). The spectra were recorded at 25°C. The solution details are specified in Materials and Methods. Three repeated experiments were carried out with similar results. The spectra presented in the figure are the visualization of one repetition.

The intrinsic fluorescence spectra demonstrated the shift of maximum fluorescence from 334±2 nm (AltMV CP contains 2 Trp and 3 Tyr residues) to 344±2 nm (AltMV SP_V_ and SP_VLP_) (Fig 5). The maximum wavelength shifts for AltMV SP_V_ and SP_VLP_ indicate that Trp and Tyr residues were located in the more hydrophilic environment in SP. The similar transition of the fluorescence maximum to a longer wavelength region had been observed previously, in the context of TMV [7] and PVX SP [6].

The ThT assay showed the presence of cross-β-structure SP_VLP_ and SP_V_ (S4 Fig), which is consistent with data for SP from TMV [7] and PVX [6]. Interestingly, the fluorescence intensity for SP_V_ was twice as high as that of SP_VLP_. This indicates, indirectly, that SP_V_ from virions contain more cross-β-structure than SP_VLP_, which is consistent with data obtained by CD spectroscopy (Table 1). It might be suggested that this result may be related to differences in the structure of AltMV virions and VLPs. As demonstrated earlier, using cryoelectron microscopy and the trypsin test, AltMV virions and VLPs have structural differences, despite their morphological similarity [4].

**Fig 6:**
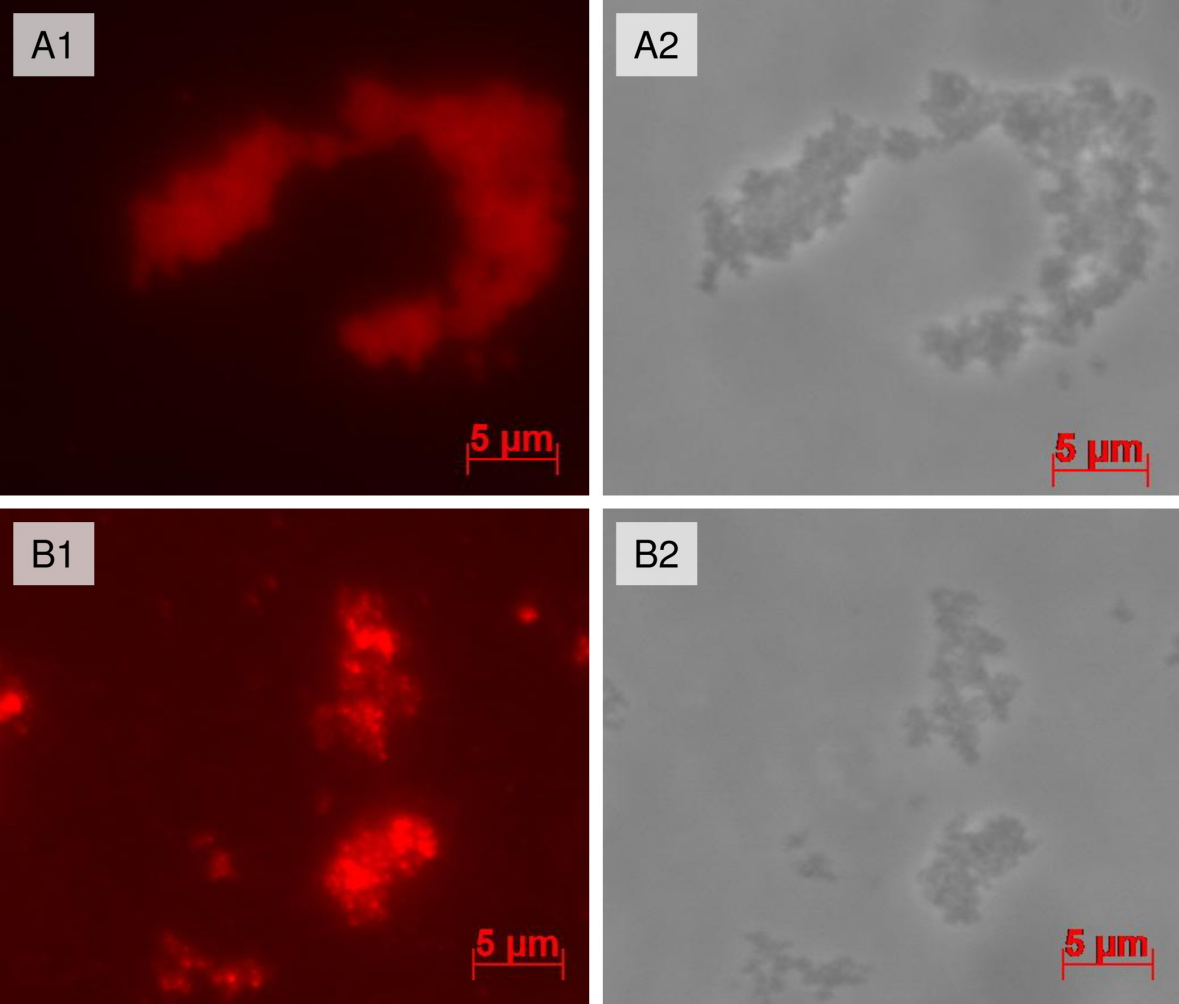
Immunofluorescence microscopy of compositions comprising SP with recombinant protein TM2e adsorbed to their surface. **A)** Complexes with SP_V_: 1.–fluorescence image. 2.–the same image in phase contrast. **B)** Complexes with SP_VLP_: 1.–fluorescence image. 2.–the same image in phase contrast. Detection with primary antibodies and secondary antibodies conjugated with fluorophore Alexa 546.

Plant viruses are an exceptionally attractive and versatile tool for biotechnology because of their biosafety for mammals. One of the attractive properties of plant virus-based SP is their ability to bind target molecules on the surface [5,6,9,11,27–29]. In the present work, preliminary data have shown that AltMV SP_V_ and SP_VLP_ could also be used as a platform for biotechnological purposes. To demonstrate adsorption properties of AltMV SP_V/VLP_, complexes with the model antigen were obtained (recombinant protein TM2e containing repeated sequences of influenza virus A M2e epitope, MW 24 kDa). SP_V_–TM2e and SP_VLP_–TM2e complexes were prepared by simple mixing with a mass ratio of 10:1 and they were analysed by immunofluorescence microscopy (Figs 6 and S5).

The fluorescence images of both complexes (Fig 6A1 and 6B1) correspond to the phase contrast images (Fig 6A2 and 6B2). Comparing the images, it can be concluded that all SP in the camera field are covered by the model antigen. This also indicates that the model antigen preserves antigenic specificity within complexes. The absence of fluorescence in the negative control samples (S5 Fig), which were not treated with primary antibodies, confirmed that fluorescence-detected binding was specific. Therefore, it has been established that both SP_V_ and SP_VLP_ form complexes with target protein by non-covalent adsorption *in vitro*.

**Fig 7:**
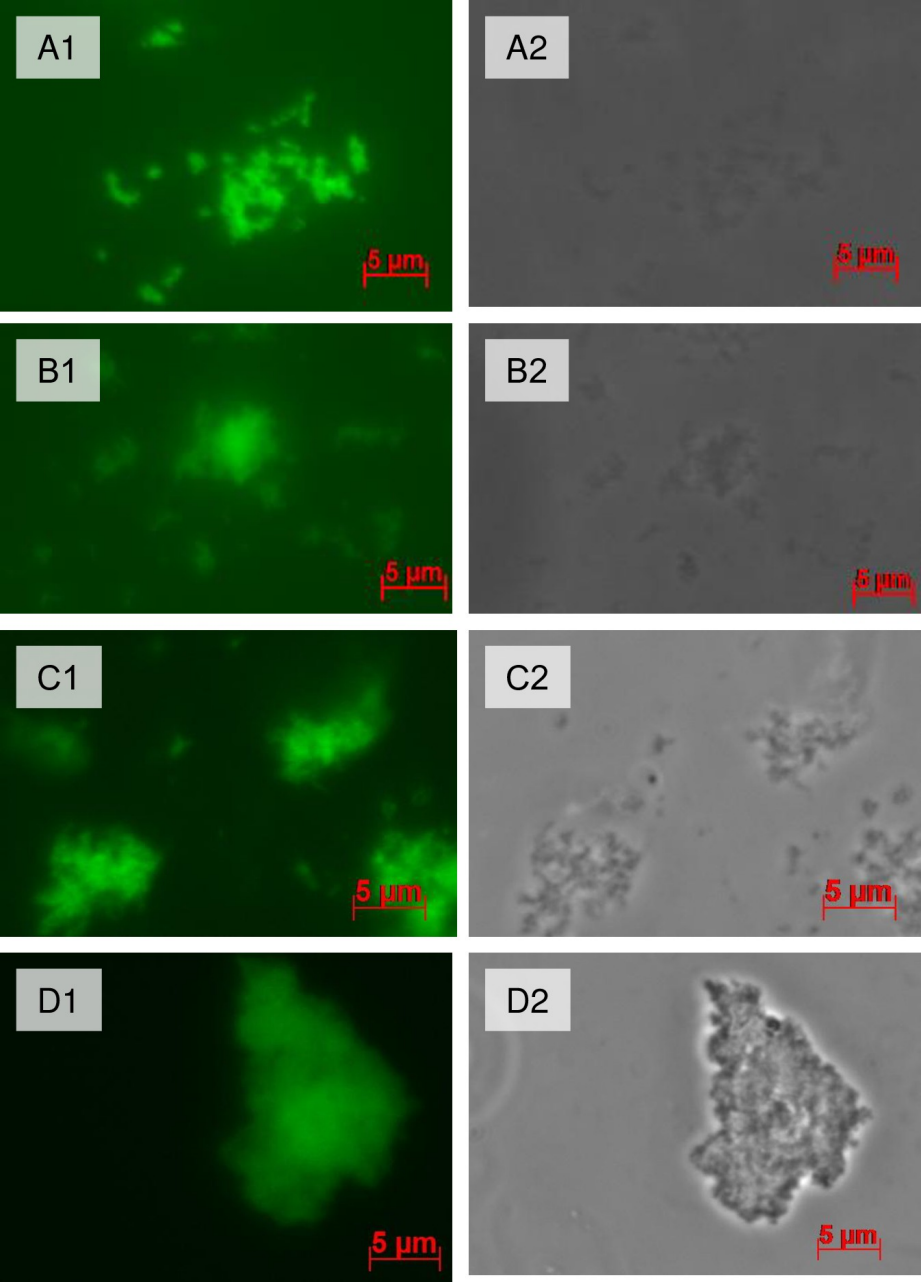
AltMV SP fluorescence labelling. **A)** SP_V_ bind FITC. **B)** SP_VLP_ bind FITC. **C)** SP_VLP_ bind 5-(N-Maleimido)-fluorescein diacetate. **D)** SP_V_ bind 5-(N-Maleimido)-fluorescein diacetate. 1 –fluorescence image, 2 –phase contrast image.

To continue to characterise the biotechnological potential of AltMV SP, surface amino and thiol groups’ reactivity was studied by using Fluorescein isothiocyanate (FITC) and 5-(N-Maleimido)-fluorescein diacetate. AltMV CP contains eight Lys which could bind FITC and two Cys potentially available to 5-(N-Maleimido)-fluorescein diacetate. SP_V_ and SP_VLP_ were labelled using two dyes and the particles obtained were studied using fluorescence microscopy (Fig 7). According to the results, both fluorophore molecules were successfully incorporated into the AltMV SP_V_ and SP_VLP_.

SDS-PAGE electrophoresis, with subsequent visualisation using UV-light (S6 Fig), demonstrated that AltMV VLPs and both types of SP interact with Fluorescein isothiocyanate (FITC) and 5-(N-Maleimido)-fluorescein diacetate considerably effective than AltMV virions. This means that Lys and Cys amino acid residues that are not available on virions’ surface become exposed on AltMV VLPs, SP_V_ and SP_VLP_. Comparing the fluorescence intensity of labelled virions and VLPs it can be concluded that CP within virions and VLPs possess different surface amino acids composition. Thus, we completed our earlier results [4] obtained using the indirect method of trypsin test. It demonstrated the alteration of trypsin hydrolysis sites presented on the surface of AltMV virions and VLPs.

The thermal denaturation of AltMV virions probably led to the appearance on SP_V_ surface amino acids capable of reacting with FITC and 5-(N-Maleimido)-fluorescein diacetate. The same conclusions had been reached for TMV virions and TMV SP labelled by FITC [12]. These data suggest that AltMV VLPs, SP_V_ and SP_VLP_ are attractive for amino groups’ labelling/conjugation. Herewith, AltMV SP_V/VLP_ is more promising than virions and VLPs for thiol labelling/conjugation.

Thus, the possibility of SP_V_ formation from AltMV virions has been described for the first time. Differences in the thermal remodelling of AltMV virions and VLPs were detected. AltMV virions transformed into SP_V_ *via* intermediate forms, as demonstrated for TMV and PVX. AltMV VLPs converted to SP_VLP_ without analogous intermediates. The temperature of the complete structural transition of AltMV virions was 94°C, and for VLPs it was 65°C. According to the biochemical analysis, SP_V_ did not contain viral RNA. Therefore, both SP_V/VLP_ are exclusively protein particles. Differences in the secondary and tertiary structures of CP within untreated virions, VLPs and SP_V/VLP_, were revealed. The intrinsic fluorescence spectra demonstrate that Trp and Tyr residues are located in the more hydrophilic environment in SP_V/VLP_. CP within SP_V/VLP_ contained more β-structures and fewer α-helixes than virions and VLPs. This is a reflection of SP_V/VLP_ formation being accompanied by significant changes in secondary and tertiary structures. The possibility of bioconjugation reactions targeted at Lys and Cys amino acid residues in AltMV SP_V/VLP_ has been demonstrated. In this way, AltMV SP_V/VLP_ represent protein platforms with exposed on their surface Lys/Cys amino acids. This makes it possible to apply AltMV SP_V/VLP_ as a base for the formation of functionally active complexes through conjugation with surface reactive amino acid residues or through non-specific adsorption.

## Supporting information

S1 Fig Selection of conditions for AltMV SPV formation. **A)** PBS, pH 7.4; **B)** Milli-Q; **C)** 0.01 M Tris-HCl, pH 8.0; **D)** 0.001 M citrate buffer, pH 4–4.2; **E)** phosphate buffer, pH 7.4; **F)** 0.15 M NaCl. Thermal treatment at 98°C for 30 sec. Transmission electron microscopy, staining with 2% uranyl acetate. Bars, 1μm.
(TIF)

S2 Fig Mean diameter of AltMV SPV with initial concentrations of 0.1, 1, 2 and 5 mg/ml. Summary data from four separate samples of SP_V_ for each concentration. Error bars represent standard deviation. Statistical differences were analysed using one-way ANOVA with a *post hoc* Tukey HSD Test, **p <0.01.
(TIF)

S3 Fig Characterisation of protein state in AltMV SPV/VLP. **1)** Protein molecular weight markers, kDa; **2) 3)** AltMV virions; **4)** AltMV SP_V_; **5)** AltMV VLPs; **6)** AltMV SP_VLP_. 8–20% SDS-PAGE, staining with Coomassie G-250.
(TIF)

S4 Fig Thioflavin fluorescence spectra of AltMV virions (1), VLPs (3), SPV (2) and SPVLP (4) with an initial concentration of 1 mg/ml. Spectra were recorded at 25°C. The solution details are specified in Materials and Methods. Three repeated experiments were carried out with similar results. The spectra presented in the figure are the visualization of one repetition.
(TIF)

S5 Fig Negative controls to the experiment presented in Fig 6. The samples were prepared in the same way as in the experiment (see Fig 6) but the primary antibodies were not added. **A)** Complexes with SP_V_: 1.–Fluorescence image. 2.–the same image in phase contrast. **B)** Complexes with SP_VLP_: 1.–Fluorescence image. 2.–the same image in phase contrast. Immunofluorescence microscopy. Secondary antibodies conjugated with fluorophore Alexa 546.
(TIF)

S6 Fig AltMV CP fluorescence labelling. **A)** Labelling by FITC. AltMV within the following structures: lane 3 –AltMV virions, lane 4 –AltMV VLPs, lane 5 –AltMV SP_V_, lane 6 –AltMV SP_VLP_. Lane 1 –protein molecular weight markers, kDa, lane 2 –unlabelled AltMV virions. 8–20% SDS-PAGE. **A1)** Staining with Coomassie G-250, **A2)** visualisation in UV-light. **B)** Labelling by 5-(N-Maleimido)-fluorescein diacetate. AltMV CP included in the following structures: lanes 2,3 –AltMV VLPs, lanes 4,5 –AltMV SP_VLP_, lanes 6,7 –AltMV virions, lanes 8,9 –AltMV SP_V_. **B1)** Staining with Coomassie G-250, **B2)** visualisation in UV-light.
(TIF)

S1 Raw images (PDF)
