# Supplementary figures and images for: Thermal remodelling of Alternanthera mosaic virus virions and virus-like particles into protein spherical particles
Source: PLoS One. 2021 Jul 28;16(7):e0255378. doi: 10.1371/journal.pone.0255378 (PMC8318239; doi:10.1371/journal.pone.0255378)

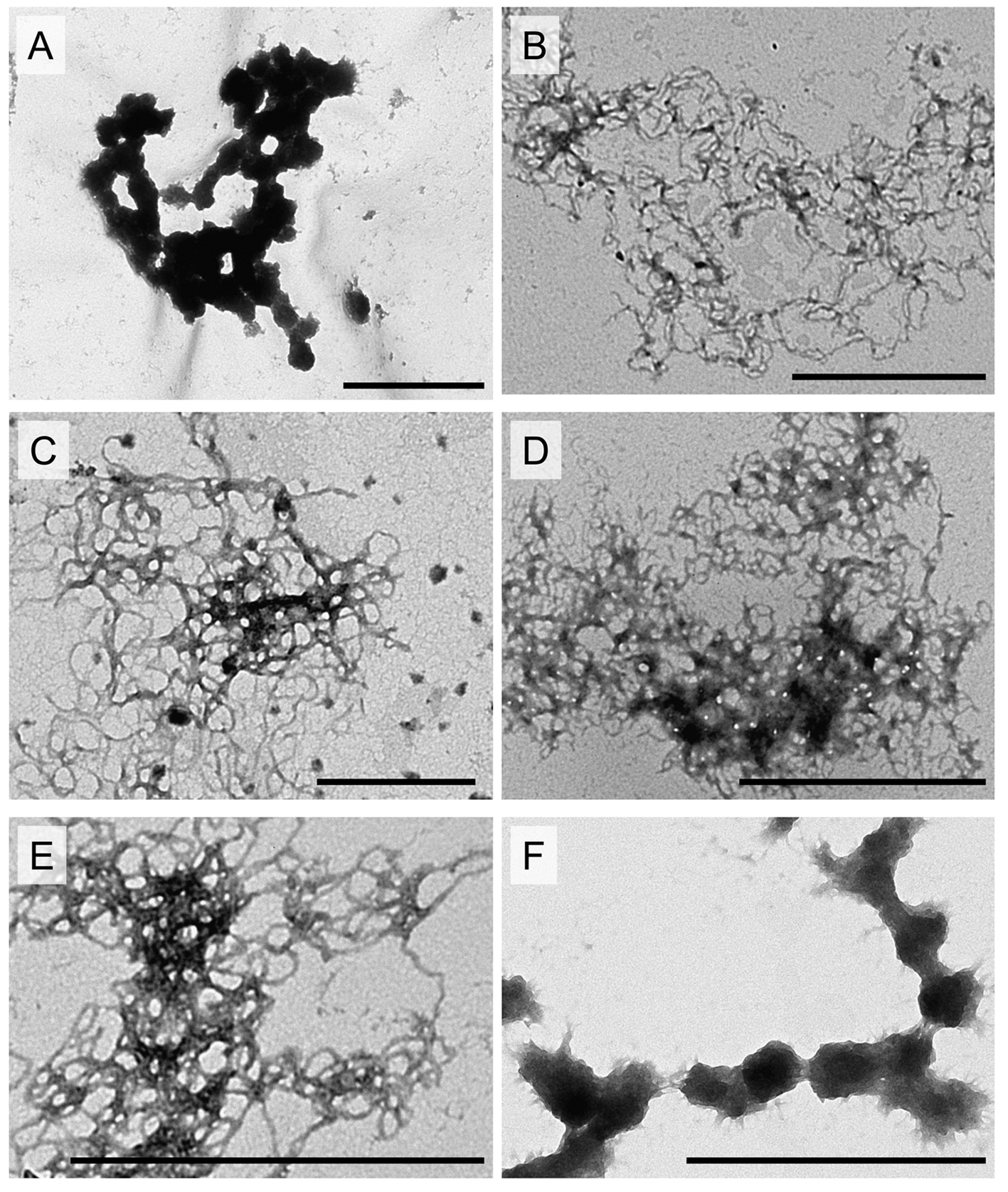

Supplement: S1 Fig — A) PBS, pH 7.4; B) Milli-Q; C) 0.01 M Tris-HCl, pH 8.0; D) 0.001 M citrate buffer, pH 4–4.2; E) phosphate buffer, pH 7.4; F) 0.15 M NaCl. Thermal treatment at 98°C for 30 sec. Transmission electron microscopy, staining with 2% uranyl acetate. Bars, 1μm. (TIF) [file pone.0255378.s001.tif]

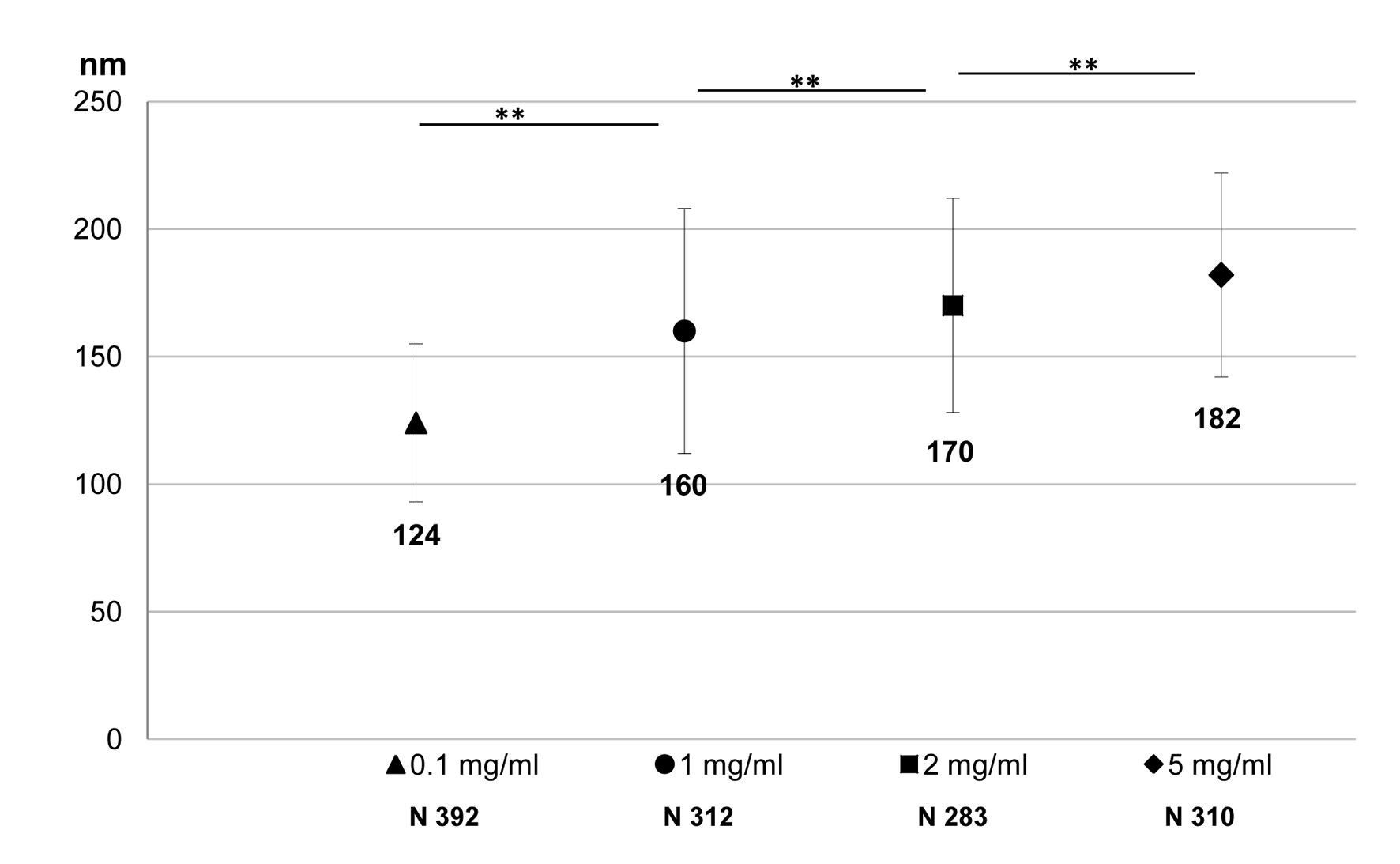

Supplement: S2 Fig — Summary data from four separate samples of SPV for each concentration. Error bars represent standard deviation. Statistical differences were analysed using one-way ANOVA with a post hoc Tukey HSD Test, **p <0.01. (TIF) [file pone.0255378.s002.tif]

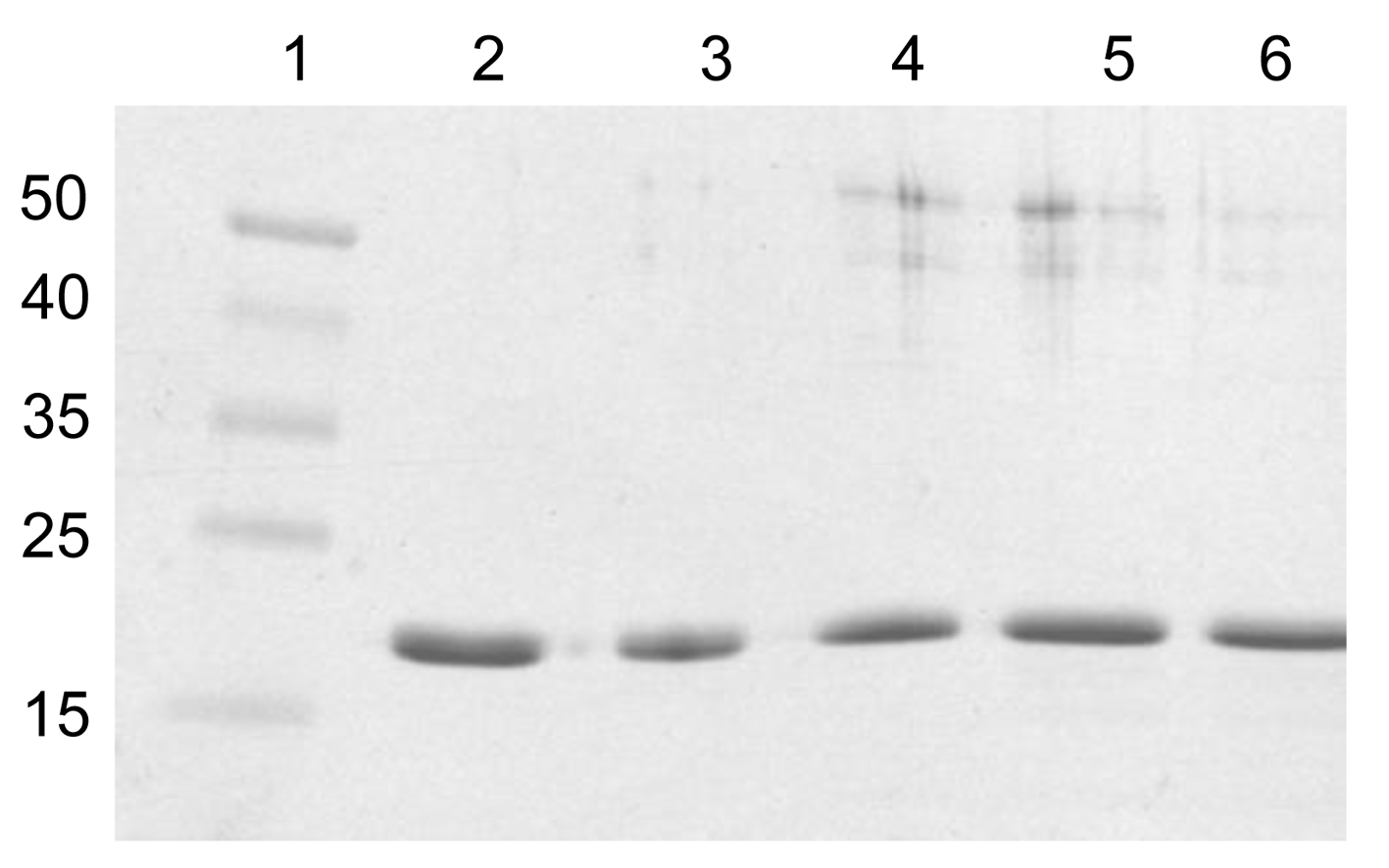

Supplement: S3 Fig — 1) Protein molecular weight markers, kDa; 2) 3) AltMV virions; 4) AltMV SPV; 5) AltMV VLPs; 6) AltMV SPVLP. 8–20% SDS-PAGE, staining with Coomassie G-250. (TIF) [file pone.0255378.s003.tif]

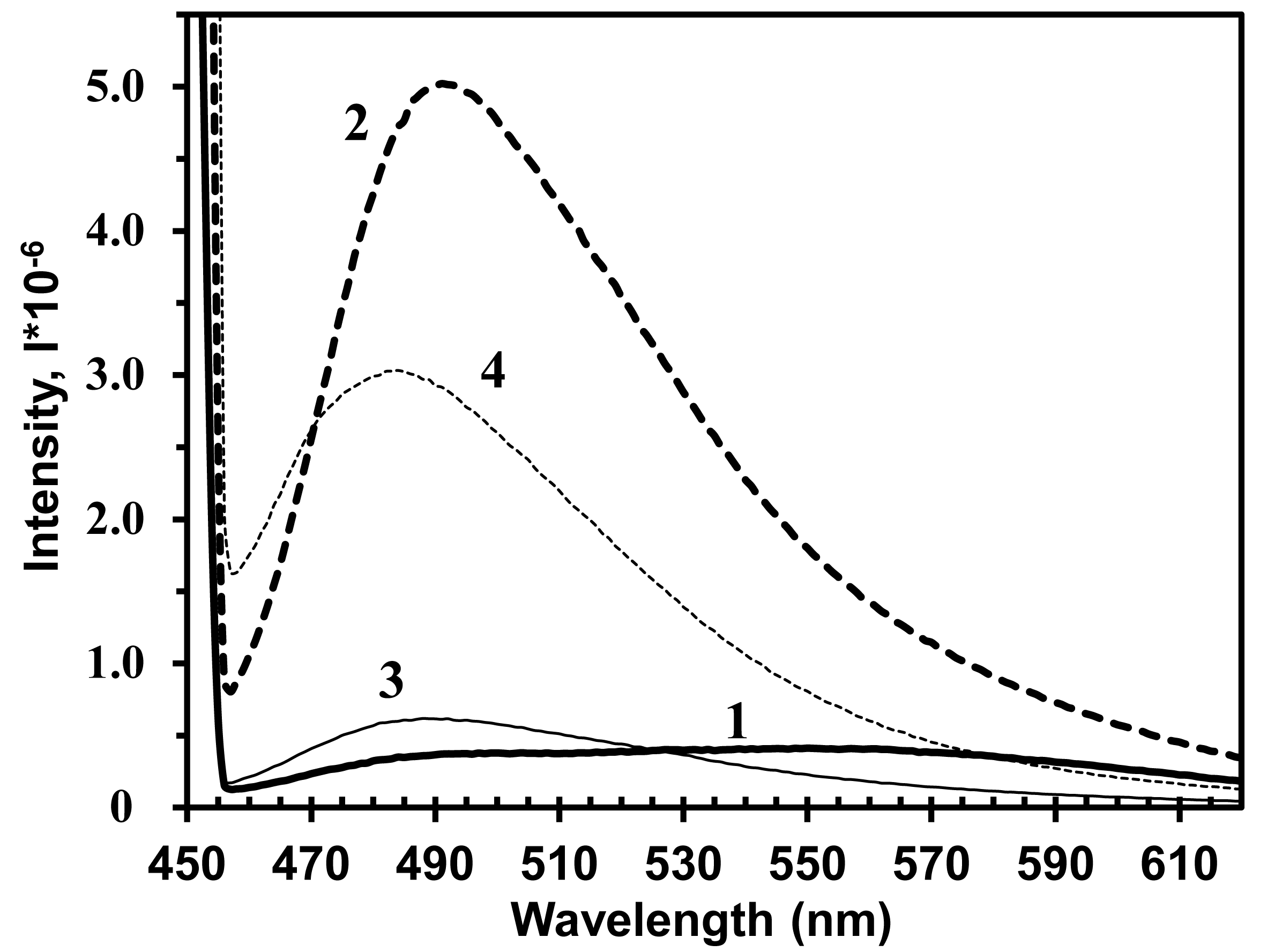

Supplement: S4 Fig — Spectra were recorded at 25°C. The solution details are specified in Materials and Methods. Three repeated experiments were carried out with similar results. The spectra presented in the figure are the visualization of one repetition. (TIF) [file pone.0255378.s004.tif]

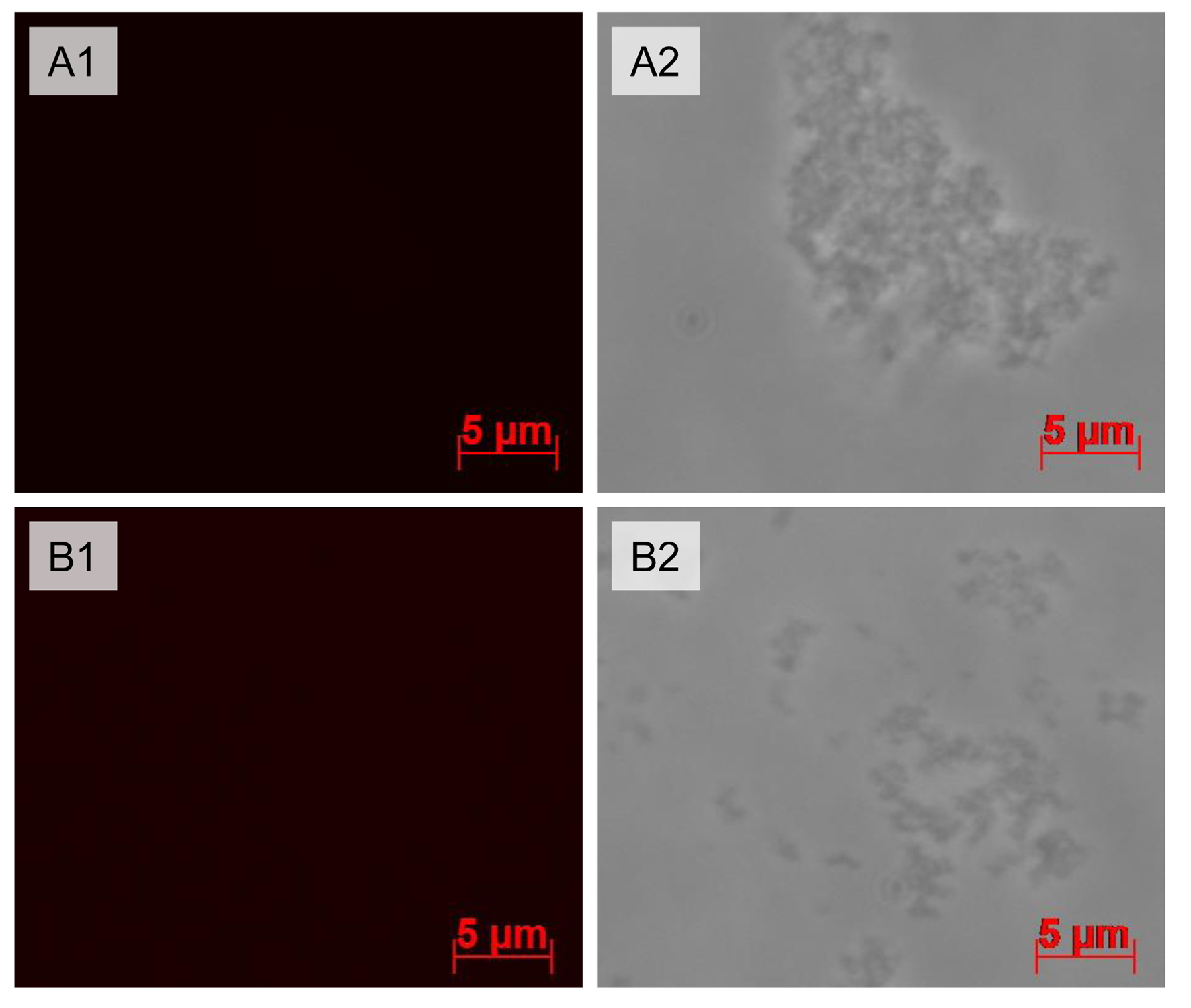

Supplement: S5 Fig — The samples were prepared in the same way as in the experiment (see Fig 6) but the primary antibodies were not added. A) Complexes with SPV: 1.–Fluorescence image. 2.–the same image in phase contrast. B) Complexes with SPVLP: 1.–Fluorescence image. 2.–the same image in phase contrast. Immunofluorescence microscopy. Secondary antibodies conjugated with fluorophore Alexa 546. (TIF) [file pone.0255378.s005.tif]

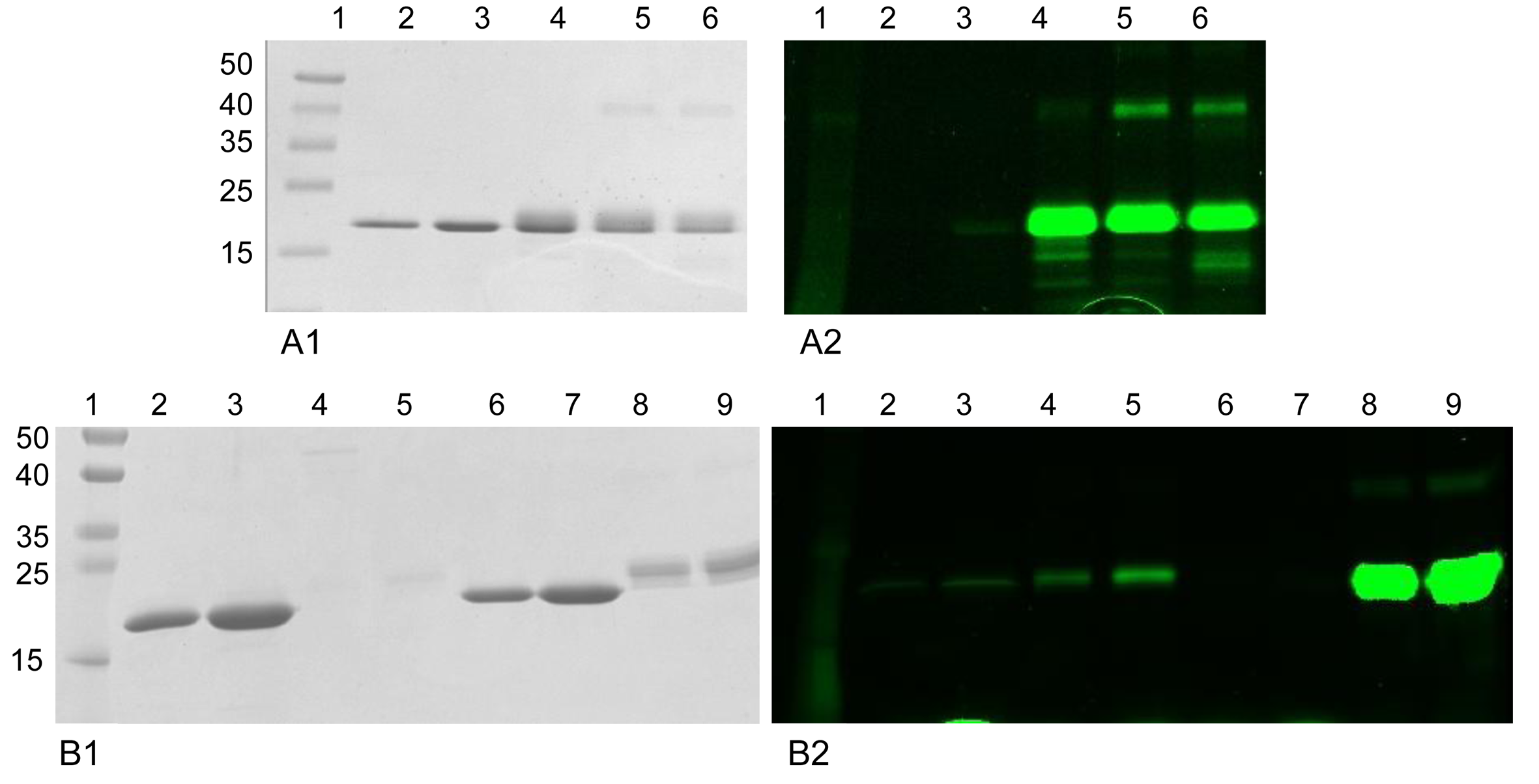

Supplement: S6 Fig — A) Labelling by FITC. AltMV within the following structures: lane 3 –AltMV virions, lane 4 –AltMV VLPs, lane 5 –AltMV SPV, lane 6 –AltMV SPVLP. Lane 1 –protein molecular weight markers, kDa, lane 2 –unlabelled AltMV virions. 8–20% SDS-PAGE. A1) Staining with Coomassie G-250, A2) visualisation in UV-light. B) Labelling by 5-(N-Maleimido)-fluorescein diacetate. AltMV CP included in the following structures: lanes 2,3 –AltMV VLPs, lanes 4,5 –AltMV SPVLP, lanes 6,7 –AltMV virions, lanes 8,9 –AltMV SPV. B1) Staining with Coomassie G-250, B2) visualisation in UV-light. (TIF) [file pone.0255378.s006.tif]
