# Supplementary material for: Thermal remodelling of Alternanthera mosaic virus virions and virus-like particles into protein spherical particles
Source: PLoS One. 2021 Jul 28;16(7):e0255378. doi: 10.1371/journal.pone.0255378 (PMC8318239; doi:10.1371/journal.pone.0255378)

Original image for Fig 3

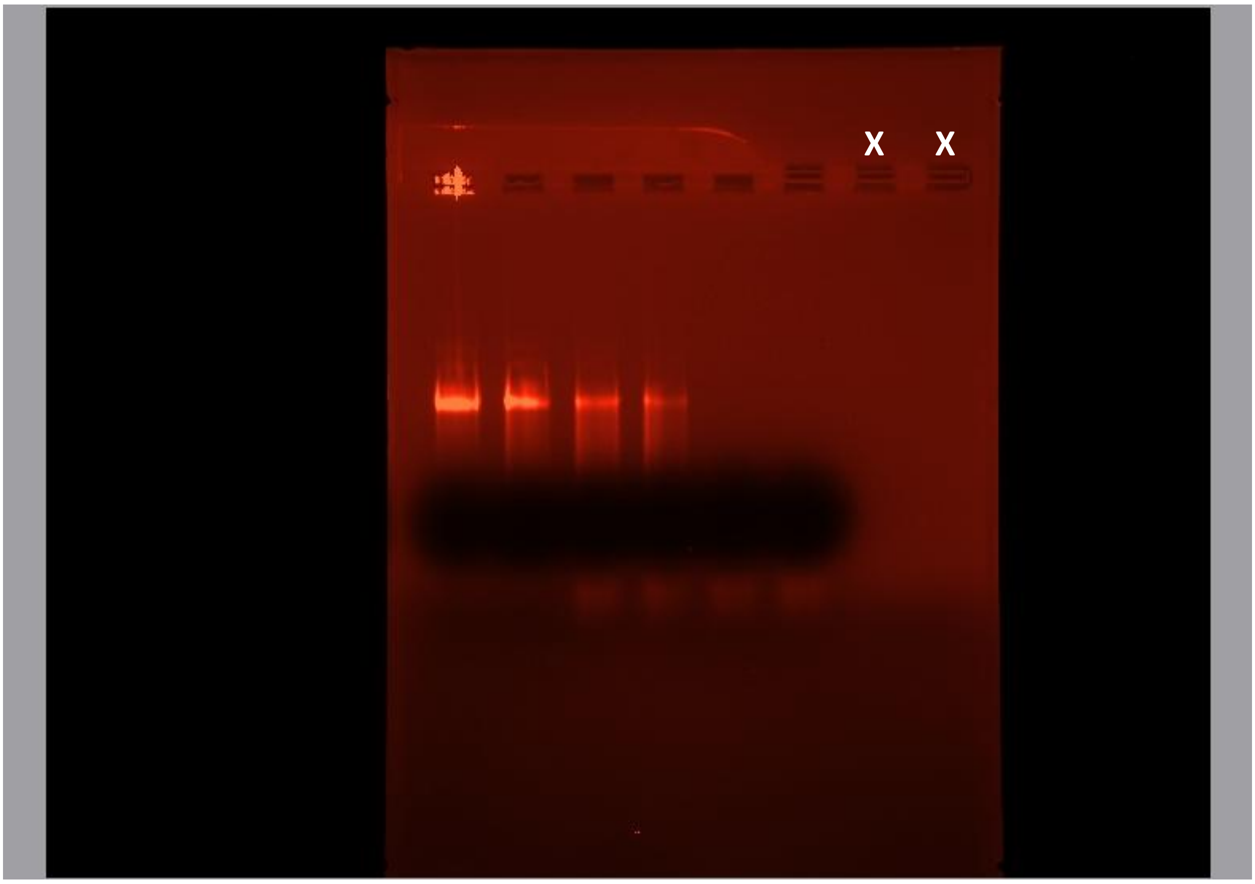

Original image for S3 Fig

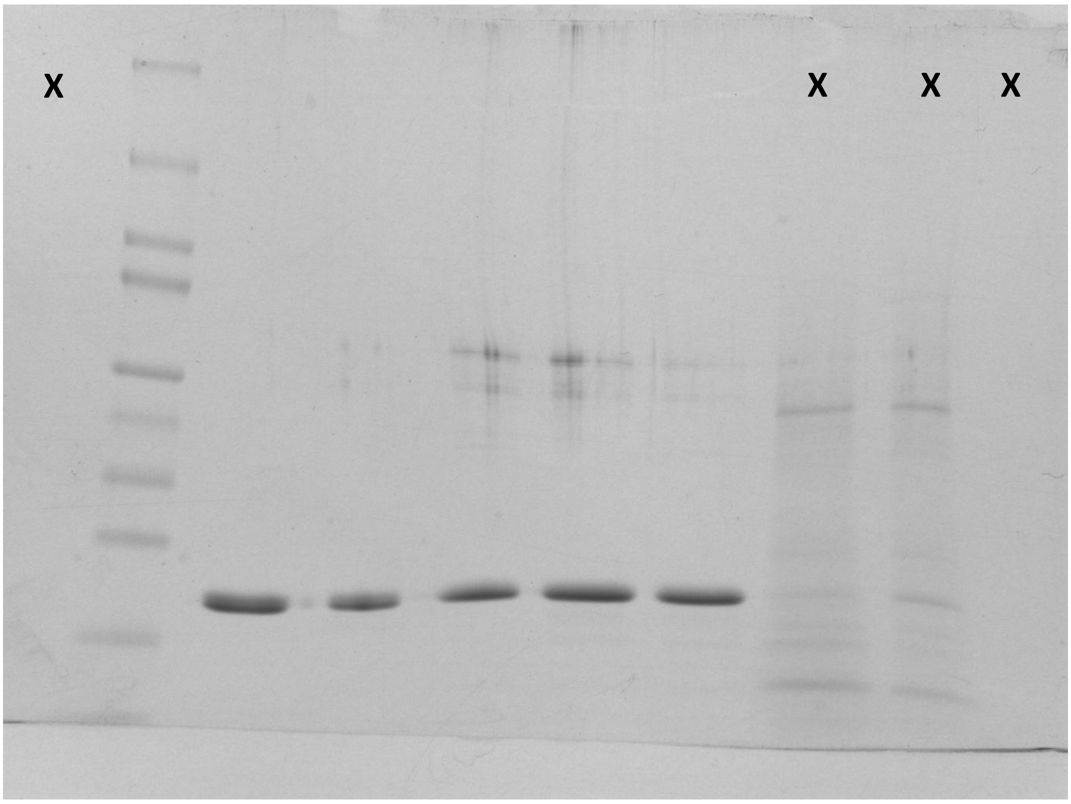

Original image for S6A1 Fig

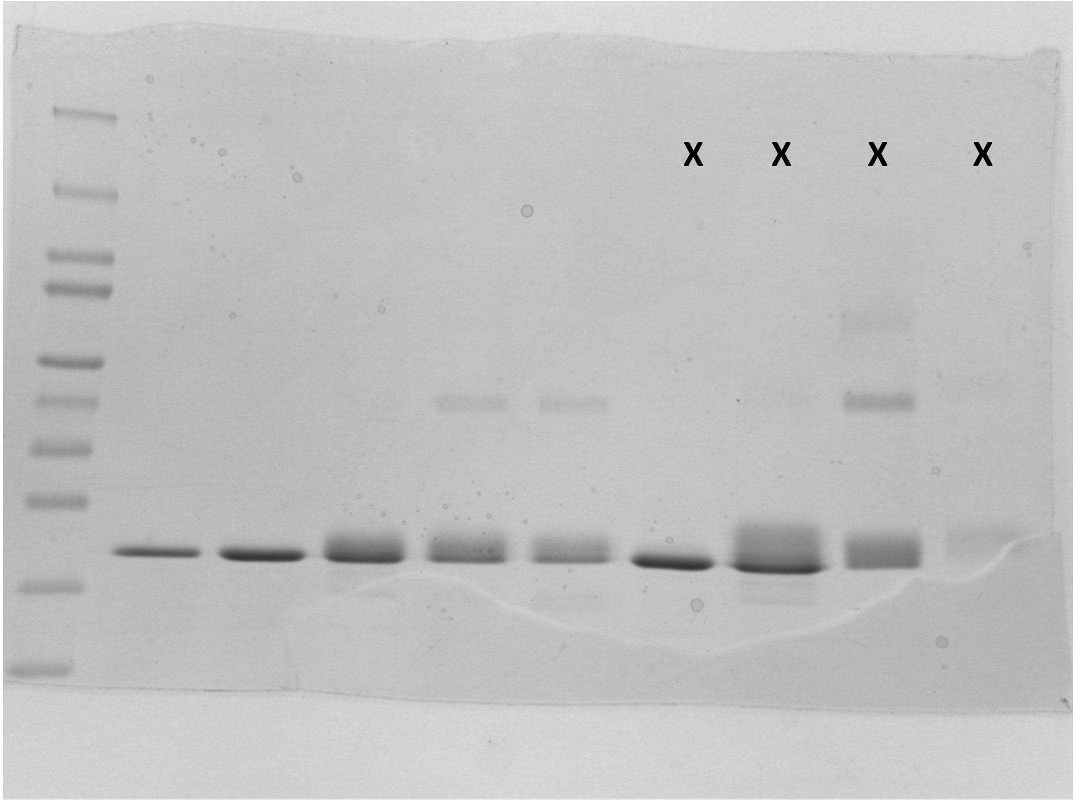

Original image for S6A2 Fig

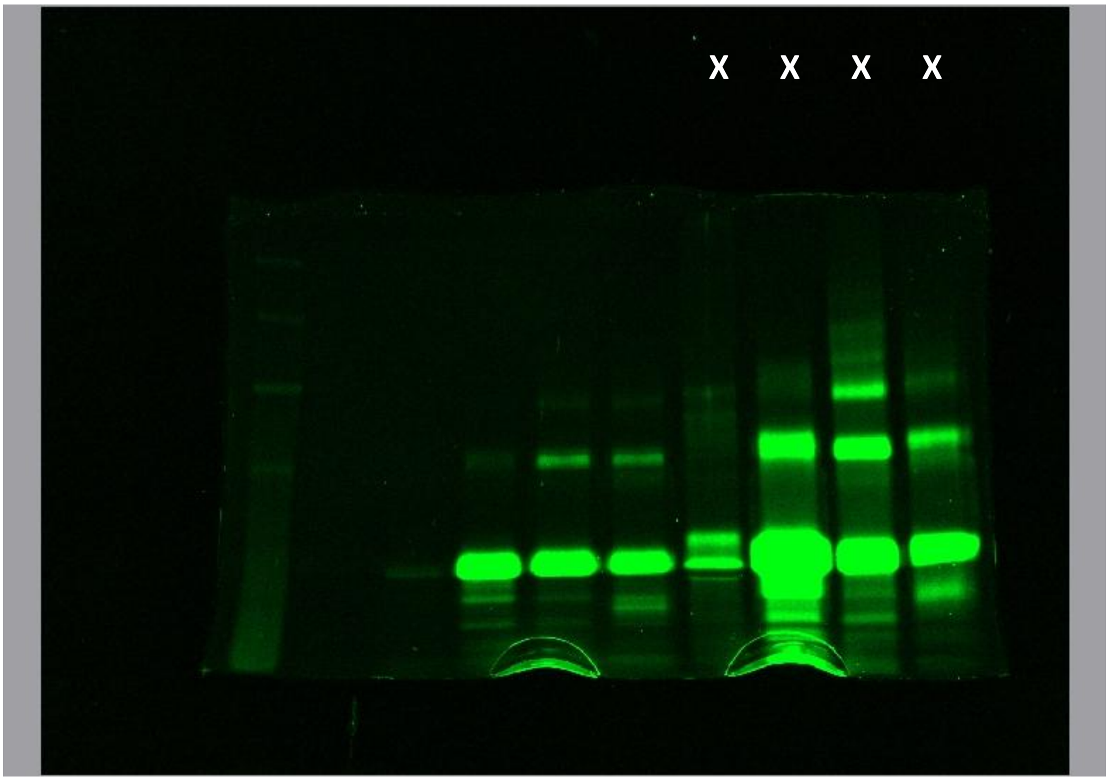

Original image for S6B1 Fig

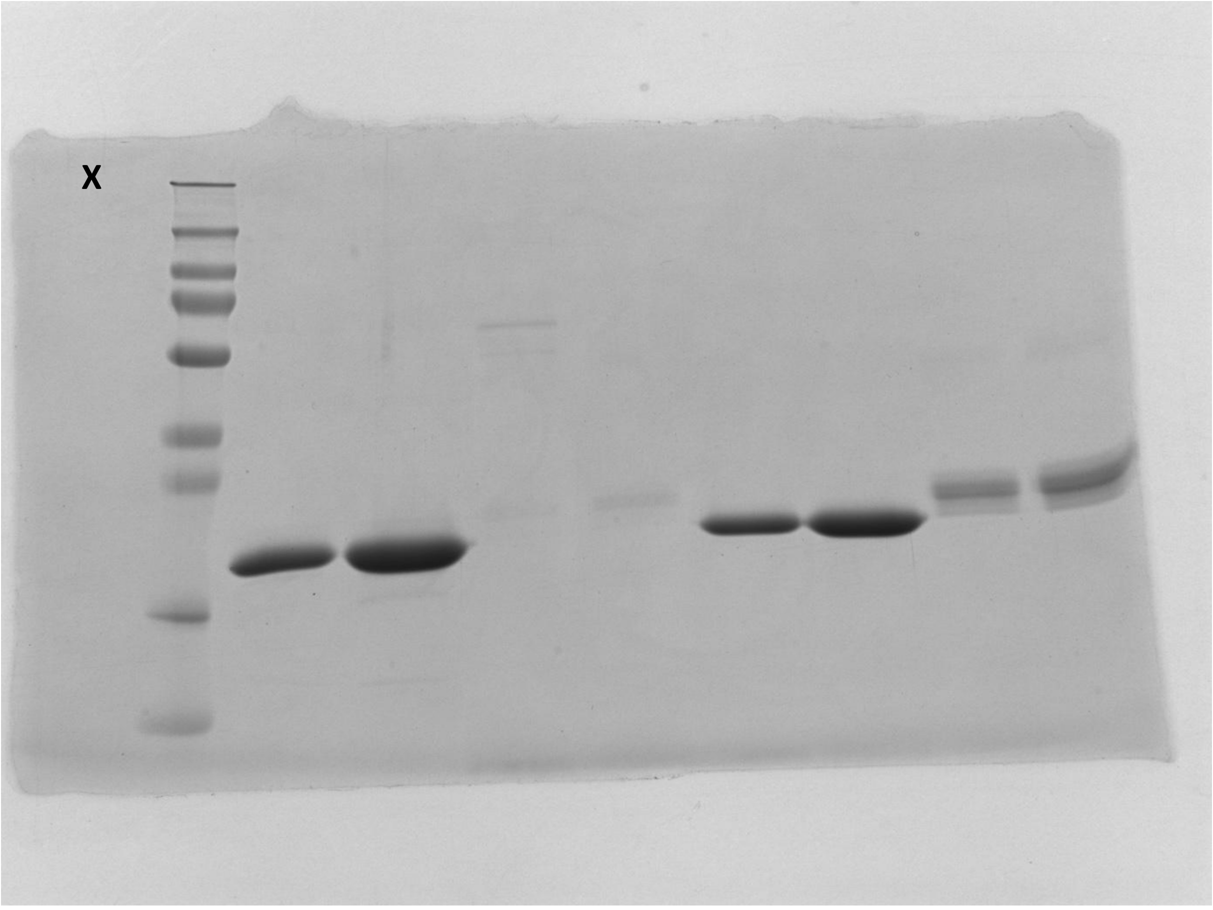

Original image for S6B2 Fig

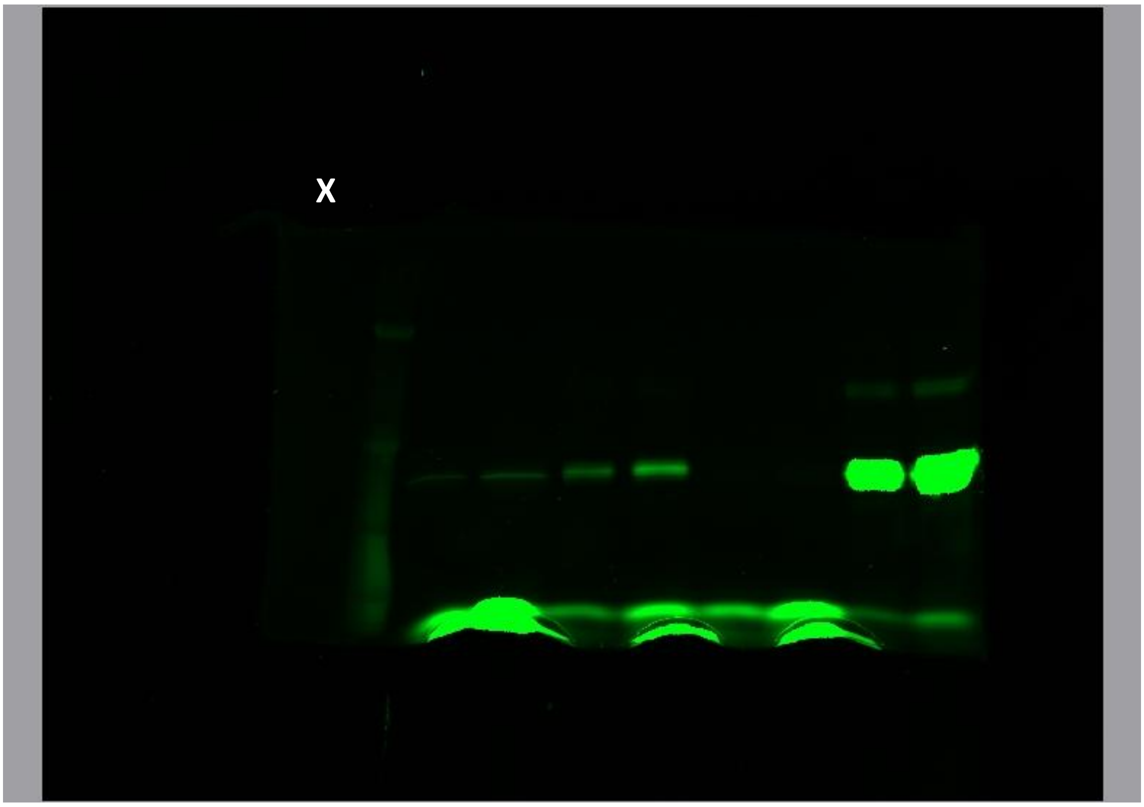

Supplement: S1 Raw images — (PDF) [file pone.0255378.s007.pdf]
